# Tobacco smoking as a risk factor for tuberculous pleural effusion: a case-control study

**DOI:** 10.1017/gheg.2020.1

**Published:** 2020-02-12

**Authors:** Pavit Tewatia, Rajeev Mohan Kaushik, Reshma Kaushik, Sanjeev Kumar

**Affiliations:** 1 Department of General Medicine, Himalayan Institute of Medical Sciences, Swami Rama Himalayan University, P.O. Jolly Grant- 248016, Dehradun, Uttarakhand, India; 2 Department of Pulmonary Medicine, Himalayan Institute of Medical Sciences, Swami Rama Himalayan University, P.O. Jolly Grant- 248016, Dehradun, Uttarakhand, India

**Keywords:** India, risk factor, smoking, tobacco, tuberculous pleural effusion

## Abstract

This study assessed the tobacco smoking-associated risk for tuberculous pleural effusion (TPE) in India. Ninety-two patients with TPE and 184 controls were randomly selected and assessed regarding their tobacco-smoking status and type, quantity and duration of tobacco used. Odds ratios (ORs) for the association of smoking cigarette, beedi and cigarette or beedi with TPE were 19.22 (*p* < 0.0001), 2.89 (*p* = 0.0006) and 4.57 (*p* < 0.0001) respectively. ORs for developing TPE increased with an increase in beedi/cigarette consumption, duration and pack years of smoking (*p* < 0.001 each). TPE was significantly associated with confounding risk factors viz., regular alcohol use (OR = 1.89, *p* = 0.019), history of contact with tuberculosis (TB) patient (OR = 8.07, *p* < 0.0001), past history of TB (OR = 22.31, *p* < 0.0001), family history of TB (OR = 9.05, *p* = 0.0002) and underweight (OR = 3.73, *p* = 0.0009). Smoking (OR = 3.07, *p* < 0.001), regular alcohol use (OR = 2.10, *p* = 0.018), history of contact with TB patient (OR = 4.01, *p* = 0.040), family history of TB (OR = 10.80, *p* = 0.001) and underweight (OR = 5.04, *p* < 0.001) were independently associated with TPE. Thus, both cigarette- and beedi-smoking have a significant association with TPE. The risk for TPE in tobacco smokers is dose- and duration-dependent.

## Introduction

Tuberculosis (TB) is the leading cause of mortality by an infectious disease [1]. More than 9 million active and new TB cases are diagnosed annually, accounting for more than 2 million deaths annually worldwide [2].

Tuberculous pleural effusion (TPE) is a frequently seen clinical condition and is regarded as a form of extra-pulmonary tuberculosis (EPTB) [3]. A pleural effusion is seen in about 5% of patients with TB [4]. High protein and glucose concentrations are seen in effusions which are predominantly lymphocytic in nature and are small to moderate in size [5].

An important preventable risk factor for the development of TB is smoking tobacco [4]. Increased risk of TB in smokers may be due to various factors like reduction in natural killer cytotoxic activity, suppression of T cell function in both lung and blood, impairment of mucociliary clearance of particles and increase in the number of alveolar macrophages in the lower respiratory tract. Immediate or innate immunity is affected by cells of the macrophage-phagocytic group as they handle and eliminate mycobacteria. As such cigarette smoke may impair the macrophage and dendritic cell function leading to persistence and/or replication of ingested mycobacteria [6–9].

Tobacco is consumed in various forms including smoking by nearly 20% of the world's population [4]. It has been seen that where multinational companies have expanded their markets, there is increased consumption of cigarettes with a high prevalence of TB [10, 11]. As India is a developing country, tobacco use in the form of beedis – a thin, Indian cigarette, made of flaked tobacco wrapped in a dried tendu (*Diospyros melanoxylon*) leaf is common, particularly by people belonging to lower socioeconomic classes [12]. The recent studies have found weak legal infrastructure to curb or regulate tobacco use in endemic areas of TB [4, 13].

A strong association exists between smoking and TB [12]. The risk of latent TB is estimated to increase by 1.9 times, active TB two times and risk of death by TB 2.6 times in smokers after adjusting for the socioeconomic status [14]. There is a dose response relationship with respect to the duration of smoking and the quantity of cigarettes [12]. As smoking is highly prevalent, these smoking-related moderate increases in individual risk of TB might have a large impact on the population level. For example, in India, 38% of deaths as a result of TB in middle aged men are attributed to smoking, with an adverse impact on the Indian economy [14, 15].

Various studies have shown contradictory findings regarding risk for EPTB in smokers [12, 16, 17]. Moreover, there is no study assessing smoking as a risk factor exclusively for TPE. Therefore, this study was carried out to determine the tobacco smoking-associated risk for TPE in a referral hospital in Dehradun, India.

## Methods

This case-control study was conducted in the Department of General Medicine, Himalayan Institute of Medical Sciences, Dehradun, Uttarakhand, India from June, 2017 to June 2018. Ethical clearance for the study was taken from the institutional ethics committee.

A sample size of 92 cases with TPE and 184 age- and sex-matched controls was estimated for detecting significant association between TPE and smoking at a power of 80% and a 5% level of significance, assuming the frequency of TPE as 0.1% in non-smokers and 7% in smokers. Patients above the age of 18 years who were admitted in medicine/chest wards of the Himalayan Institute Hospital, Dehradun, India with a primary diagnosis of TPE were eligible for inclusion in the study. A written informed consent was taken from all study participants.

A case of TPE was defined as a patient with exudative pleural effusion with either of the following i.e., pleural fluid acid fast bacilli (AFB) smear positive, or polymerase chain reaction/cartridge based nucleic acid amplification test (PCR/CBNAAT) positive for TB, or pleural biopsy showing necrotic caseous granuloma, or responding to antituberculous treatment (ATT) in the form of improvement of symptoms. Patients with congestive heart failure, chronic liver disease, chronic kidney disease, underweight, diabetes, malignancy or any concomitant disease known to cause pleural effusion, patients who were human immunodeficiency virus (HIV) positive with acquired immunodeficiency syndrome and patients with concomitant pulmonary tuberculosis (PTB) or disseminated TB were excluded from the study.

Patients presenting with symptoms of cough, shortness of breath, chest pain, with or without fever and weight loss, with evidence of pleural effusion on examination or chest X-ray, were subjected to detailed history, clinical examination and evaluation for tuberculous etiology. Cases underwent routine investigations including complete haemogram, erythrocyte sedimentation rate (ESR), urine examination, biochemical profile (liver function tests, fasting plasma glucose, blood urea nitrogen, serum creatinine, serum lactate dehydrogenase [LDH]), sputum smear examination for AFB for two consecutive days by Ziehl Neelsen (ZN) staining, sputum culture for AFB (Lowenstein-Jensen culture media) (wherever indicated), chest X-ray, electrocardiogram (ECG), screening for HIV (HIV comb immunoassay), Mantoux test, pleural fluid analysis: total white cell count, differential white cell count, total protein and glucose, adenosine deaminase (ADA), LDH, ZN staining for AFB, AFB culture, cytology for malignant cells, CBNAAT (GX4 gene Xpert MTB/Rif test system) and PCR test for TB (wherever required). Ultrasound examination of thorax/abdomen and pleural biopsy was done, if required.

Cases and controls were selected by simple random sampling using a random number table. Patients with predefined exclusion criteria were excluded before random sampling. Out of 387 cases diagnosed to have TPE, 92 cases were selected for the study. The selection of controls was started after selecting the required number of cases to allow for the age- and sex-matching. Two age- and sex-matched controls were taken for each case. Overall, 184 controls were selected out of 2537 patients admitted in the hospital who did not have symptoms suggestive of TB and had undergone routine haematological and biochemical investigations, testing for HIV, chest X-rays and ECG for their ailments and were proved as not having TB or other diseases falling under exclusion criteria for cases.

Baseline characteristics of cases and controls were tabulated and compared. Baseline characteristics considered were age, gender, area of residence, socioeconomic status, overcrowding at home, high risk occupation, smoking, regular alcohol use, history of contact with TB patient, past history of TB, family history of TB and underweight. A detailed information about smoking status, duration of smoking, age at which smoking started, type of tobacco smoked and quantity of beedis or cigarettes smoked/day were recorded from cases and controls. Pack years of smoking were used to quantify the person as mild (1–10 pack years), moderate (11–20 pack years) or heavy (>20 pack years) smoker.

A smoker was defined as a person who smoked any tobacco product, either daily or occasionally. A pack year of smoking was calculated by multiplying the number of packs of cigarettes/beedis smoked per day by the number of years the person had smoked [18].

### Statistical analysis

Results were analysed by SPSS (version 20.0). Data were presented in the form of percentages, proportions and means. Baseline characteristics of cases and controls were compared using the χ^2^ test or Fisher's exact test for qualitative data and Student's unpaired *t*-test for quantitative data.

Odds ratios (ORs) were estimated for measuring the effect. The χ^2^ test of association was used for testing the association between TPE and various variables (area of residence, low socioeconomic status, overcrowding at home, high risk occupation, smoking status and type, dose, duration and pack years of smoking, regular alcohol use, history of contact with TB patient, past history of TB, family history of TB and underweight) about which information was recorded from subjects at the time of their entry into the study. Variables found to be statistically significant i.e. smoking, regular alcohol use, history of contact with TB patient, past history of TB, family history of TB and underweight were assessed as potential independent predictors by multivariate analysis using the logistic regression model. Goodness of fit for the logistic regression model was determined by Cox and Snell R square.

## Results

Baseline characteristics of cases and controls are shown in Table 1. The age range of cases was 18–95 years. Male preponderance was seen in cases (78.2%). The maximum number of cases as well as controls belonged to low and middle socioeconomic status. Among cases, 72.8% were smokers and the majority of the smokers were males. Twenty-nine (31.5%) cases consumed cigarettes and 38 (41.3%) consumed beedis. Among controls, 36.9% were smokers who were mostly males. Seven (3.8%) controls smoked cigarettes and 61 (33.1%) smoked beedis. The number of smokers was significantly higher among cases than controls (*p* < 0.0001). The mean age of starting smoking was significantly less (*p* = 0.002) while the mean number of cigarettes/beedis smoked per day (*p* = 0.0001) and pack years of smoking (*p* = 0.011) were significantly higher among cases than controls. Regular alcohol use, history of contact with TB patient, past history of TB, family history of TB and underweight were present in a significantly higher number of cases than controls (*p* < 0.05 each). No significant differences were seen regarding area of residence, socioeconomic status, overcrowding at home, high risk occupation and mean duration of smoking between cases and controls (*p* > 0.05 each).

**Table 1.** Baseline characteristics of cases (*n* = 92) and controls (*n* = 184)^a^

Characteristics	Cases *n* (%)	Controls *n* (%)	Cases *v.* controls *p* value
Age group (years)			
18–30	23 (25)	46 (25)	0.887
31–45	26 (28.2)	52 (28.2)	0.887
46–60	18 (19.5)	36 (19.5)	0.862
61–75	19 (20.6)	38 (20.6)	0.887
76–90	6 (6.5)	12 (6.5)	0.791
Mean age (years)	49.9 ± 19.0	48.12 ± 18.5	0.455
Gender			
Male	72 (78.2)	144 (78.2)	0.887
Female	20 (21.7)	40 (21.7)	0.887
Area of residence			
Rural	69 (75.0)	117 (63.5)	0.076
Urban	23 (25.0)	67 (36.4)	0.076
Socio-economic status			
High^b^	1 (1)	1 (0.5)	1
Middle^c^	42 (45.6)	88 (47.8)	0.823
Low^d^	49 (53.2)	95 (51.6)	0.887
Overcrowding at home^e^	34 (36.9)	61 (33.1)	0.624
High risk occupation	3 (3.2)	2 (1.0)	0.337
Smokers			
Total	67 (72.8)	68 (36.9)	<0.0001
Male	50 (54.3)	52 (28.2)	1
Female	17 (18.4)	16 (8.6)	1
Mean age for starting smoking (years)	17.3 ± 4.4	19.7 ± 7	0.002
Type of smoking			
Cigarette	29 (31.5)	7 (3.8)	<0.0001
Beedi	38 (41.3)	61 (33.1)	<0.0001
Number of cigarettes/beedis smoked per day	14.2 ± 6.6	10.61 ± 4.39	0.0001
Duration of smoking (years)	27.9 ± 14.6	30.56 ± 11.9	0.106
Smoking pack years	19.46 ± 12.62	16.01 ± 9.52	0.011
Regular alcohol use	44 (47.8)	60 (32.6)	0.019
History of contact with TB patient	14 (15.2)	4 (2.1)	<0.0001
Past history of TB	10 (10.8)	1 (0.5)	<0.0001
Family history of TB	12 (13.0)	3 (1.6)	0.0002
Underweight^f^	19 (20.6)	12 (6.5)	0.0009

TPE, tuberculous pleural effusion; TB, tuberculosis.

a Data are number (%) of patients or mean ± s.d.

b Annual family income >Rs. 850 000 (1 Pound Sterling = 92.90 Indian Rupees).

c Annual family income Rs. 50 000–850 000.

d Annual family income <Rs. 50 000.

e Expressed as the number of persons per room exceeding the accepted standards of 2 persons in 1 room, 3 in 2 rooms, 5 in 3 rooms, 7 in 4 rooms and 10 persons in 5 rooms (an additional 2 for each further room).

f Body mass index <18.5 kg/m^2^.

The clinical profile of patients with TPE is shown in Table 2. The majority of patients had left sided pleural effusion (57.6%), followed by right sided (32.6%) and bilateral pleural effusion (9.7%). Pleural fluid was exudative in all cases and showed predominantly lymphocytic cells. The unadjusted OR for the development of TPE due to smoking was found to be 4.57 and a strong association was seen between tobacco smoking and TPE (*p* < 0.0001). Cigarette-smoking (*p* < 0.0001) as well as beedi-smoking (*p* = 0.0006) had a significant association with TPE. The OR for the development of TPE rose consistently with an increase in the number of beedis/cigarettes consumed. The OR of 2.57 was recorded in cases smoking beedis/cigarettes 1–10/day and was statistically significant (*p* = 0.004) whereas OR was 12.26 in cases who smoked cigarettes/beedis 11–20/day and was highly significant (*p* < 0.0001). A highly significant association was seen between the number of cigarettes/beedis smoked per day and TPE (*p* < 0.0001). A significant association was seen between smoking duration and TPE (*p* < 0.001). A highly significant OR of 5.70 was observed in cases who had smoking duration of greater than 20 years (*p* < 0.0001). The OR for TPE showed a consistent rise with an increase in the pack years of smoking. The ORs of 3.77, 4.09 and 5.17 were observed in cases having pack years of smoking 1–10, 11–20 and >20 respectively and were highly significant (*p* < 0.01 each). A highly significant association was present between pack years of smoking and TPE (*p* < 0.0001) (Table 3).

**Table 2.** Clinical profile and laboratory parameters of cases (*n* = 92)

Clinical feature	*n* (%)^a^
Symptoms	
Fever	66 (71.7)
Shortness of breath	60 (65.2)
Weight loss	42 (45.6)
Cough	39 (42.3)
Chest pain	34 (36.9)
Others	3 (3.2)
Signs	
Pallor	52 (56.5)
Oedema	32 (34.7)
Clubbing	14 (15.2)
Hepatomegaly	13 (14.1)
Icterus	4 (4.3)
Lymphadenopathy	2 (2.1)
Cyanosis	1 (1.0)
Laboratory parameters	
Haemoglobin (g/dL)	11.77 ± 2.17
WBC count (thousand/cumm)	10.13 ± 8.42
ESR (mm/h)	51.7 ± 14.8
Raised ESR	81 (88)
Pleural fluid	
WBC count (cells/cumm)	422.2 ± 216.5
Sugar (mg/dL)	80.49 ± 43.50
Protein (g/dL)	4.5 ± 1.1
Raised ADA	78 (84.7)
Raised LDH	86 (93.4)
AFB positive by ZN staining	3 (3.2)
AFB culture positive	28 (30.4)
CBNAAT positive	29 (31.5)
PCR test positive for TB	40 (43.4)
Necrotic caseous granuloma shown by thoracoscopic pleural biopsy	12 (13.0)

WBC, white blood cell; ESR, erythrocyte sedimentation rate; ADA, adenosine deaminase; LDH, lactate dehydrogenase; AFB, acid fast bacilli; ZN, Ziehl Neelsen; CBNAAT, cartridge based nucleic acid amplification test; PCR, polymerase chain reaction; TB, tuberculosis.

a Data are number (%) of patients or mean ± s.d.

**Table 3.** Association between TPE and smoking and risk of TPE as per type, dose, duration and pack years of smoking

Group	Number of smokers	Number of non-smokers	Smokers									
			Type of smoking		Number of cigarettes/beedis smoked/day		Smoking duration (years)			Pack years of smoking		
			Beedi smoker	Cigarette smoker	1–10	11–20	1–10	11–20	>20	1–10	11–20	>20
Cases	67	25	38	29	30	37	13	11	43	13	15	39
Controls	68	116	61	7	54	14	19	14	35	16	17	35
Odds ratio	4.57		2.89	19.22	2.57	12.26	3.17	3.64	5.70	3.77	4.09	5.17
95% CI	2.64–7.91		1.59–5.22	7.57–48.80	1.38–4.79	5.78–26.00	1.38–7.26	1.48–8.96	3.06–10.60	1.61–8.81	1.80–9.27	2.75–9.69
*p* value	<0.0001		0.0006	<0.0001	0.004	<0.0001	0.006	0.005	<0.0001	0.002	<0.001	<0.001
χ^2^	30.16		17.13		15.78		12.34			32.33		
*p* value	<0.0001		<0.0001		<0.0001		<0.001			<0.0001		

TPE, tuberculous pleural effusion; CI, confidence interval.

Association of TPE with various confounding factors was also analysed. Of these, regular alcohol use (*p* = 0.019), history of contact with TB patient (*p* < 0.0001), past history of TB (*p* < 0.0001), family history of TB (*p* = 0.0002) and underweight (*p* = 0.0009) showed a significant association with TPE. Other confounding risk factors such as low socio-economic status (*p* = 0.887), overcrowding (*p* = 0.624) and high risk occupation (*p* = 0.337) did not show a statistically significant association with TPE (Table 4). Smoking, regular alcohol use, history of contact with TB patient, past history of TB, family history of TB and underweight having significant association with TPE were examined for their independent association with TPE by multivariate analysis using logistic regression. Cox and Snell R square was used to determine the Goodness of fit for the logistic regression model and was 0.47. Logistic regression analysis showed that smoking (*p* < 0.001), regular alcohol use (*p* = 0.018), history of contact with TB patient (*p* = 0.040), family history of TB (*p* = 0.001) and underweight (*p* < 0.001) had an independent association with TPE (Table 5).

**Table 4.** Univariate analysis showing association between various confounding risk factors and TPE

Variables	Odds ratio	95% CI	*p* value
Area of residence (rural *v.* urban)	1.71	0.98–3.00	0.076
Low socioeconomic status	1.06	0.64–1.76	0.887
Overcrowding	1.18	0.70–1.99	0.624
High risk occupation	3.06	0.50–18.68	0.337
Regular alcohol use	1.89	1.13–3.16	0.019
History of contact with TB patient	8.07	2.57–25.31	<0.0001
Past history of TB	22.31	2.81–177.23	<0.0001
Family history of TB	9.05	2.48–32.95	0.0002
Underweight	3.73	1.72–8.08	0.0009

TPE, tuberculous pleural effusion; TB, tuberculosis.

**Table 5.** Multivariate analysis showing independent risk factors for TPE

Variables	*B*	s.e.	Wald	df	Sig.	Exp (*B*)
Smoking	1.123	0.317	12.539	1	<0.001	3.073
Regular alcohol use	0.742	0.314	5.589	1	0.018	2.101
History of contact with TB patient	1.390	0.676	4.225	1	0.040	4.017
Past history of TB	1.750	0.899	3.790	1	0.052	5.752
Family history of TB	2.380	0.711	11.215	1	0.001	10.801
Underweight	1.617	0.452	12.832	1	<0.001	5.040

TPE, tuberculous pleural effusion; TB, tuberculosis; B, coefficient for the constant; s.e., standard error; Wald, Wald χ^2^ value; df, degrees of freedom; Sig., significance probability or *p* value; Exp (B), exponentiation of the B coefficient.

Variables entered in the logistic regression model: smoking, regular alcohol use, history of contact with TB patient, past history of TB, family history of TB and underweight; ‘Smoking’ as a variable includes either beedi or cigarette smoking.

## Discussion

Mycobacterium TB infection is found in one third of the world's population. Among them, active TB develops in 9 million every year, causing death in 2 million annually [19]. Our study was conducted to find the risk of TPE among smokers.

We observed that most of the cases were middle aged men. The mean age was 49.9 ± 19 years. Male preponderance (78.2%) was seen in cases and also in smokers (50/67, 74.6%). The mean age at which cases started smoking was 17.3 ± 4.4 years. The mean duration of smoking seen in our cases was 27.9 ± 14.6 years. In a study conducted by Gambhir *et al*., similar age trend was seen in the same geographical area. The mean age in their cases was 43.2 ± 16.7 years and male preponderance was also seen in their study. The mean age at which cases started smoking was 22.1 ± 6 years [12].

Most (98.9%) of the patients included in our study belonged to low (53.2%) and middle (45.6%) socioeconomic status. This could have affected their knowledge and awareness regarding TB due to low levels of education, leading to increased prevalence of TB in this segment of population. Similarly, in another study conducted by Ozturk *et al*., 28.1% people belonged to the category having low income (<200 euro/month) [20].

The majority (56.7%) of smokers among our cases were beedi smokers while 43.2% smoked cigarettes. High prevalence of beedi smoking may be due to the low and middle socioeconomic status of our cases. Our findings are supported by findings of Singh *et al*., that in India, beedi smoking and dual use of beedi and cigarette were inversely associated with wealth while cigarette smoking was directly associated with wealth. Moreover, cigarette smoking was directly associated with education [21].

The mean number of cigarettes/beedis smoked per day was 14.2 ± 6.6 among cases. The duration of smoking was 1–10 years in 19.4%, 11–20 years in 16.4% and >20 years in 64.1% cases. In a study by Gambhir *et al*., 7.3% cases were heavy smokers and the mean number of beedis/cigarettes smoked per day was 15 ± 6.87. The duration of smoking was more than 20 years in 61.9% cases [12]. In another study, quantity of smoking was < 20 pack years in 45% of smokers and >20 pack years in 55% [20]. In our study too, quantity of smoking was more than 20 pack years in 58.2% of smokers among cases. Moreover, the mean value of smoking pack years was significantly higher in cases than controls, implying that smoking cigarettes/beedis for a considerable period of time increases the risk of TPE.

Various studies have shown prevalence of alcohol abuse ranging from 10.4% to 99% among smokers [12, 16, 22, 23]. Almost half of our cases were using alcohol on a regular basis. As 72.8% of our cases were smokers, many of them were exposed to risks due to the concomitant use of alcohol and smoking.

A total of 20.6% of our cases were underweight similar to the results of another study, showing 15.7% of TB cases as underweight [12]. However, Nijenbandring de Boer *et al*. reported this figure as 62.9% among their TB cases [23].

Over-crowding at home was present in 36.9% of our cases. In a study from India, over-crowding was seen in 31.5% of TB cases [12]. In another study conducted in Istanbul, over-crowding was observed in 41.1% of TB cases [20]. Overcrowding at home in a sizeable number of TPE cases implies that it is an important risk factor for TPE as it facilitates the spread of TB bacteria leading to the development of TPE in at least some people who contract TB infection.

In our study, history of contact with TB patient was present in 15.2%, past history of TB in 10.8% and family history of TB in 13.0% cases. High risk occupation was seen in 3.2% cases. Our findings are in agreement with findings of another study which showed a positive family history of TB in 11.5% cases, history of contact with TB patient in 15.7%, past history of TB in 16.8% and high risk occupation in 2.1% cases [12]. It suggests that a past history of TB or a prolonged or short term contact with TB patients could make one more prone to develop TPE due to reactivation of TB or as a sequel to primary infection.

In our study, the main presenting symptoms were fever (71.7%), shortness of breath (65.2%), weight loss (45.6%), cough (42.3%) and chest pain (36.9%). Our cases had predominantly left sided pleural effusion (57.6%). Another study described cough (64.6%), shortness of breath (60.7%), fever (48.1%), chest pain (35.7%), expectoration (32.4%), weight loss (18.3%), fatigue (10.2%), night sweats (6.6%) and haemoptysis (1.2%) as main symptoms in their patients having TPE [24]. The presence of pleuritic chest pain points towards the involvement of pleura in such a clinical setting.

Pleural fluid analysis was done in all cases and was exudative in nature. ZN staining for AFB was positive in 3.2% and AFB culture in 30.4% cases. The PCR test for TB was positive in 43.4% and CBNAAT in 31.5% cases. In a study conducted by Amer *et al*., AFB was demonstrated by ZN staining or AFB culture in 4% and 32% of patients respectively. They also observed 34% patients having positive PCR for TB [25].

In our study, a significant risk of TPE was observed among smokers (OR 4.57, *p* < 0.0001). Risk of TPE increased with an increase in quantity (*p* < 0.0001), duration (*p* < 0.001) and pack years of smoking (*p* < 0.0001). Risk was more with cigarette smoking (OR 19.22, *p* < 0.0001) than beedi smoking (OR 2.89 *p* = 0.0006). Standard logistic regression analysis in our study also showed smoking as an independent risk factor for TPE (OR 3.07, *p* < 0.001). Another study conducted by Ahmad *et al*., showed highly significant odds (OR 2.35, *p* < 0.01) of TB recurrence with continuation of smoking within 2 years after completing the course of ATT [19]. Other studies conducted in Taiwan [26], South Korea [27] and Iran [28] suggested that smoking leads to an increase in the risk of recurrence by 1.44, 1.99 and 2 times respectively. Feng *et al*. observed the proportions of latent tuberculosis infection (LTBI) cases in ex-smokers, current smokers and never-smokers as 39.4% (58/147), 42.4% (53/125) and 25% (151/603) respectively. LTBI was significantly lower in the never-smokers (*p* < 0.001) while both ex-smoking (OR 1.64) and current smoking (OR 1.88) were independent factors associated with LTBI [29]. In another study, smoking was found to be an independent risk factor for TB using multivariate analysis (OR 3.05, *p* = 0.000) [12]. In a study conducted by Nijenbandring de Boer *et al*., the multivariate logistic regression model showed smoking as an independent risk factor in delayed culture conversion in smokers at 60 days of ATT (OR 6.85, *p* = 0.002) [23]. A study conducted in Istanbul, showed smoking (OR 4.97, *p* = 0.0001) as well as heavy smoking (>20 pack years) (OR 0.12, *p* = 0.0001) independent risk factors for the development of TB [20]. However, another study conducted by Garcia-Rodriguez *et al*., suggests that smoking had a protective effect on EPTB [16]. Similarly, another study conducted in Nepal, showed that smoking was more likely to be associated with pulmonary TB than EPTB (OR 0.34, *p* = 0.001) [22]. However, smoking was found to be an independent risk factor for the development of TPE in our study.

Univariate analysis showed that other confounding risk factors having significant association with TPE in our study were the regular use of alcohol (*p* = 0.019), history of contact with TB patient (*p* < 0.0001), history of past TB (*p* < 0.0001), family history of TB (*p* = 0.0002) and underweight (*p* = 0.0009).

Smoking and alcohol habits have been found as independent risk factors for PTB [22 30 31]. Garcia-Rodriguez *et al*., observed a lower association of tobacco and alcohol use with EPTB than PTB [16]. A meta-analysis by Webster and Shandera in Atlanta regarding habituation to alcohol or smoking showed higher rates of extra-pulmonary disease (OR 0.28, OR 0.24), in those who were not engaged in these habits [17]. However, Nijenbandring de Boer *et al*., observed that alcohol abuse had an OR 0.22 (*p* = 0.027) for the development of TB using multivariate analysis [23]. In our study, the regular use of alcohol showed a significant risk for TPE (OR 1.89, *p* = 0.019) and was found to be an independent risk factor for the same (OR 2.10, *p* = 0.018).

In our study, we observed that having a past history of TB was an important factor and had an OR 22.31 (*p* < 0.0001). A contact with TB patient and family history of TB also had significant ORs [8.07 (*p* < 0.0001) and 9.05 (*p* = 0.0002) respectively]. A contact with TB patient and family history of TB were also found to be the independent risk factors for TPE when multivariate analysis was used using the logistic regression model (OR 4.01, *p* = 0.040 and OR 10.80, *p* = 0.001) but past history of TB did not emerge as an independent risk factor for TPE (OR 5.75, *p* = 0.052). A study conducted in Rawalpindi, Pakistan, observed a significant association of smoking with recurrence of PTB after completion of treatment with anti-tuberculous drugs (OR 2.35, *p* < 0.01) [19]. Another study by Gracia-Rodriguez *et al*., showed that there was preceding TB contact in 27.5% (57/207) cases of pleural TB [16].

We observed that 20.6% cases with TPE were underweight and there was a significant association between underweight and TPE (*p* = 0.0009). Moreover, underweight emerged as an independent risk factor for the development of TPE using the logistic regression model (OR 5.04, *p* < 0.001). Poor nutrition has been observed to contribute to protein energy malnutrition and deficiencies of micronutrients [32]. Severe underweight is the most common cause of immunodeficiency worldwide and leads to abnormalities of both the innate and adaptive immunity [33] thereby increasing the risk of development of TPE.

Thus on entering smoking along with other confounding factors associated with TPE into the logistic regression model, we found that smoking, regular alcohol use, underweight, history of contact with TB patient and family history of TB were independently associated with TPE.

Considering the fact that both tobacco smoking and TPE are common all over the globe, particularly in developing countries, our study findings that tobacco smoking increases the risk of TPE warrant an urgent attention towards the problem. Tobacco smoke suppresses phagocytic ability in macrophages in response to infection [34]. As such, smokers may be more prone to acquire primary TB and those having latent TB may develop active disease leading to increased disease burden in community. As an increase in incidence of TPE can strain the healthcare resources, efforts to increase the awareness of public regarding tobacco smoking associated risk for TPE may help in bringing down the number of tobacco smokers which can lower the risk of TPE and thus its incidence. Smoking leads to relatively poor results of TB treatment among smokers due to biological mechanisms related to smoking that impair host defences [34]. Motivating the smokers with TPE to quit smoking may improve the impaired host defences against TB and boost the response to ATT. This may be achieved through the physicians involved in the care of these patients and the effective implementation of smoking cessation programmes at the governmental as well as the community level.

## Limitations of the study

Risk factors like area of residence (rural *v.* urban), socioeconomic status, overcrowding and high risk occupation were not associated with TPE in our study, still there is a remote possibility of their role as confounders. There is the possibility of potential residual confounding, due to unmeasured additional confounding factors, or the fact that the sample size may not have been sufficient in order to detect associations between many of the other confounding variables and TPE. The impact of smoking cessation on the risk of TPE was not considered as our study was a cross-sectional study.

## Conclusions

Tobacco smoking has a strong association with TPE. Both cigarette smoking and beedi smoking have a significant association with TPE. The risk of TPE increases with dose and duration of smoking. As such spreading the awareness regarding increased risk for TPE among smokers, can indirectly decrease the burden on health care facility in the long term.
